# Combustion of Titanium–Carbon Black High-Energy Ball-Milled Mixtures in Nitrogen: Formation of Titanium Carbonitrides at Atmospheric Pressure

**DOI:** 10.3390/ma13081810

**Published:** 2020-04-11

**Authors:** Michail A. Korchagin, Dina V. Dudina, Alexander I. Gavrilov, Boris B. Bokhonov, Natalia V. Bulina, Alexey V. Panin, Nikolay Z. Lyakhov

**Affiliations:** 1 Institute of Solid State Chemistry and Mechanochemistry SB RAS, Kutateladze str. 18, Novosibirsk 630128, Russia; korchag@solid.nsc.ru (M.A.K.); gavr_sand@mail.ru (A.I.G.); bokhonov@solid.nsc.ru (B.B.B.); bulina@solid.nsc.ru (N.V.B.); lyakhov@solid.nsc.ru (N.Z.L.); 2 Novosibirsk State Technical University, K. Marx Ave. 20, Novosibirsk 630073, Russia; 3 Lavrentyev Institute of Hydrodynamics SB RAS, Lavrentyev Ave. 15, Novosibirsk 630090, Russia; 4 Department of Natural Sciences, Novosibirsk State University, Pirogova str. 2, Novosibirsk 630090, Russia; 5 Institute of Strength Physics and Materials Science SB RAS, Akademicheskiy Ave. 2/4, Tomsk 634055, Russia; pav@ispms.ru

**Keywords:** titanium, ball milling, powder, self-propagating high-temperature synthesis, carbonitride

## Abstract

In this work, titanium carbonitrides were synthesized by self-propagating high-temperature synthesis (SHS) in nitrogen. For the first time, the synthesis of titanium carbonitrides by combustion was realized in nitrogen at atmospheric pressure. The synthesis was carried out by subjecting high-energy ball-milled titanium–carbon black powder mixtures to combustion in a nitrogen atmosphere. The influence of the ball milling time on the phase composition of the products of SHS conducted in the Ti+0.3C reaction mixture was studied. It was found that the titanium–carbon black mixtures need to be milled for a certain period of time for the combustion synthesis to yield a single-phase carbonitride product.

## 1. Introduction

Titanium carbide, TiC, and titanium nitride, TiN, are promising ceramic materials owing to their high melting points, high hardness, thermal stability, and high thermal and electrical conductivities [1]. As TiC and TiN are isomorphous compounds with crystal structure of NaCl, a continuous series of TiC_y_N_1−y_ (0 ≤ y ≤ 1) solid solutions can be formed. These solid solutions are synthesized as nitrogen atoms substitute for carbon atoms in the structure of TiC. The ternary phase diagram of the Ti-C-N system can be found in ref. [2]. Using titanium carbonitrides, tungsten-free cemented carbides are being developed for applications in cutting tools, wear resistant parts, coatings, and electrodes [1,3,4]. It was shown that the mechanical properties of these materials depend on the size of the titanium carbonitride grains. 

Several synthesis methods of titanium carbonitrides have been developed: solid-state reaction in TiN+TiC mixtures, processing of TiC+Ti mixtures in a nitrogen atmosphere [5], carbothermal reduction of TiO_2_ accompanied by nitridation [5], sol–gel synthesis [6], synthesis from metal-organic reaction mixtures [5,7,8], reduction of TiCl_4_+C_3_N_3_Cl_3_ mixtures by sodium [9], and reduction of TiCl_4_+C_2_Cl_4_ mixtures by magnesium in a nitrogen atmosphere [10]. Titanium carbonitrides were found in detonation sprayed coatings obtained from a titanium powder under incomplete combustion of acetylene; in those experiments, the source of nitrogen was the carrier gas [11]. 

Self-propagating high-temperature synthesis (SHS) is another viable method for obtaining titanium carbonitrides. In refs. [12,13], the synthesis occurred at high nitrogen gas pressures; the starting reactants were mixtures of titanium and carbon powders. Single-phase carbonitrides were obtained when the pressure of nitrogen was in the 0.6–50 MPa range and previously synthesized titanium carbonitride or nitride was added to the reaction mixture. 

In recent years, attempts have been made to produce titanium carbonitrides using treatment of reaction mixtures in planetary ball mills or vibratory mills. The reaction of synthesis either proceeds in the SHS mode directly in the milling vial or is carried out slowly (mechanochemical synthesis) [8,14]. The milling vials are filled with nitrogen up to pressures between 0.6 and 0.72 MPa. Depending on the composition of the reaction mixture and the type of the mill, the synthesis requires 1–100 h of mechanical treatment. Prolonged milling leads to contamination of the powder by the material of the vials and milling bodies [8]. An overview of the existing synthesis methods of titanium carbonitrides shows that they either include multiple processing stages or require long treatment times, neither of which is technologically attractive. Solid-state reactions require the use of high-temperature equipment. High pressures in the reactors or milling vials add complexity to the experimental set-ups. 

Ball milling of powder mixtures, in which exothermic reactions can be initiated, reduces the reaction onset temperatures (see, for example, studies reported in refs. [15,16,17]). Mechanical treatment in high-energy planetary ball mills changes the morphology of the powder particles and microstructure of the composite mixture. During milling, mechanocomposites form, which are composite particles tens or hundreds of micrometers in size. In the mechanocomposites, the size of the reactant particles is reduced relative to that in the untreated mixture, the reactants are thoroughly mixed with each other, and the interfacial area between the reactants is greatly increased. During high-energy ball milling, metals are subjected to severe plastic deformation, which leads to accumulation of defects and strain in their crystalline lattices; the material accumulates a fraction of the energy delivered during milling [18]. Moreover, ball milling helps generate fresh interfaces, as oxide films present on the particle surfaces tend to break, enabling direct contact between the reactants in the ball-milled mixture. These phenomena are believed to be responsible for enhanced reactivity of the components of the reaction mixtures and reduced reaction onset temperatures. In some ball-milled compositions, the interaction starts at a temperature several hundred degrees lower than in conventional (untreated) powder mixtures. 

The goal of the present communication is to report the possibility of obtaining titanium carbonitrides by combustion of titanium–carbon black powder mixtures in nitrogen supplied at atmospheric pressure. The Ti+0.3C composition was selected as an example to demonstrate the effect of the time of high-energy ball milling on the phase composition of the combustion products.

## 2. Materials and Methods 

The reaction mixtures were prepared from a titanium powder (PTOM-2, 98.5 wt.% Ti, Polema, Tula, Russia) and a carbon black powder (PM-15, 95 wt.% C, Omsk Carbon Group, Omsk, Russia). The powder of carbon black was annealed in vacuum at 850 °C for 30 min to remove the volatile components. Ball milling of the titanium–carbon black mixtures was conducted in an AGO-2 mill (a high-energy planetary ball mill with water-cooled vials, Institute of Solid State Chemistry and Mechanochemistry SB RAS, Novosibirsk, Russia). In this mill, the volume of each vial is 160 cm^3^. Steel balls of 8 mm diameter were used. The mass of the milling balls and titanium–carbon black mixture was 200 g and 10 g, respectively. The centrifugal acceleration of the milling balls was 400 m s^−2^. In order to prevent oxidation of the powders, milling was conducted in an atmosphere of argon. The powders were loaded and unloaded in a glove box. Experiments were conducted with mixtures of the following compositions: Ti+C, Ti+0.2C, Ti+0.3C, and Ti+0.5C with a focus on the Ti+0.3C composition.

**Figure 1:**
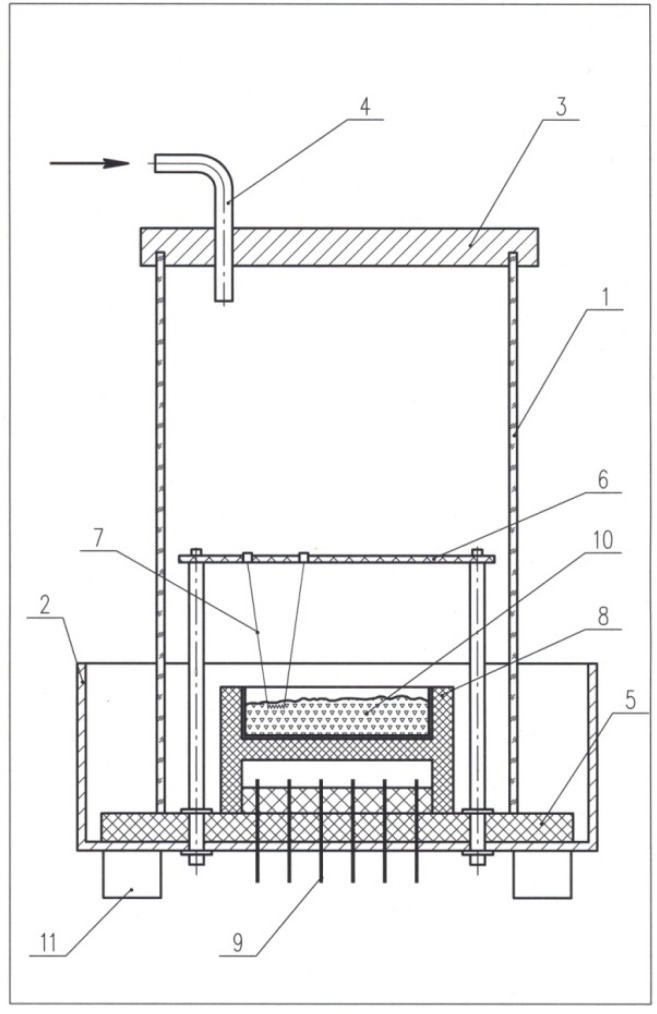
Schematic of the self-propagating high-temperature synthesis (SHS) reactor: *1* – quartz tube; *2* – tray; *3* – lid; *4* – gas sleeve; *5* – refractory plate; *6* – holder of the spiral; *7* – nichrome spiral; *8* – container; *9* – electrodes; *10* – sample; *11* – reactor stand.

The combustion reactions were carried out in a SHS reactor of continuous type having a volume of 6.6 L. The schematic of the reactor is presented in Figure 1. The main structural element of the reactor is quartz tube *1*. The diameter of the tube is 150 mm. From the top, the tube is covered with air-tight lid *3*, through which sleeve *4* is passing to enable gas supply. Stainless steel tray *2* has a diameter of 200 mm and a height of 100 mm. At the bottom of the tray, ceramic container *8* holding sample *10* is mounted. Electrode *9* enables the operation of the thermocouples and heating of igniting spiral *7*. The spiral is fixed by holder *6*. On the bottom of the tray, refractory plate *5* is placed. The thickness of the plate is 7 mm. At the bottom, the reactor is not air-tight, so the gas coming from the top is removed through the space between the tube and the refractory plate. If a gas is supplied at a rate of 9.5 L min^−1^, the excess pressure in the reactor does not exceed 10^3^ Pa. In our previous investigations, it was shown that, when an inert gas is supplied into the reactor, the combustion synthesis can be conducted without any detrimental oxidation effects on the products.

The ball-milled mixtures were cold-pressed to reach relative densities of 30%–35%. The samples were placed in a horizontal ceramic container with dimensions of 15 × 15 × 50 mm^3^. Between the walls of the container and the sample, the refractory ceramic plates were placed. The weight of the reaction mixture to be converted into the product in a single experiment was 10–12 g. 

Prior to the initiation of combustion, the reactor was flushed with nitrogen (99.996% N_2_). During the SHS and cooling of the products, nitrogen was supplied into the reactor at a flow rate of 9.5 L min^−1^. Combustion experiments were also conducted under a flow of argon (99.998% Ar) supplied into the reactor at the same flow rate.

Combustion was initiated by igniting the reaction in a B_4_C+4Ti ball-milled powder mixture (the milling time was 5 min); the weight of the ignition mixture was 0.1 g. The B_4_C+4Ti mixture was placed under a nichrome spiral (Figure 1). The spiral was heated by an electric current passing through it. An upper layer of the sample after the synthesis was mechanically removed such that no component of the reacted ignition mixture could appear in the target product of synthesis. For measuring the combustion rate and combustion temperature, W-Re thermocouples with a diameter of 100 μm were used. The thermocouples were placed at a certain distance from each other. The signals from the thermocouples were processed by an analog-to-digital converter and were transferred to a computer. 

X-ray diffraction (XRD) patterns of the ball-milled mixtures and the products of SHS were recorded by means of a D8 ADVANCE powder diffractometer (Bruker AXS, Karlsruhe, Germany) using Cu Ka radiation. The calculation of the crystallite size was carried out using the Rietveld method in TOPAS 4.2 software (Bruker AXS, Karlsruhe, Germany). The instrumental contribution to the peak width was calculated by the method of fundamental parameters [19,20].

**Figure 2:**
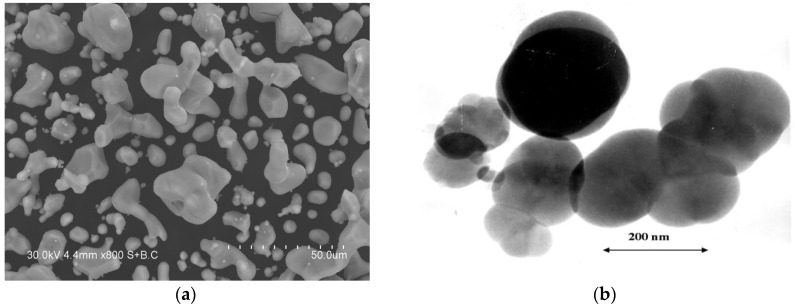
(**a**) Morphology of the titanium powder, (**b**) morphology of the carbon black powder.

The morphology of the powders was studied by scanning electron microscopy (SEM) using a Hitachi S-3400N microscope (Tokyo, Japan) and transmission electron microscopy (TEM) using a JEM 2000 FX II microscope (JEOL, Tokyo, Japan). Figure 2 shows the morphologies of the starting titanium (SEM image) and carbon black (TEM image) powders.

## 3. Results and Discussion

Titanium and carbon mixed at a molar ratio of 1:1 make one of the most exothermic SHS systems. The adiabatic combustion temperature of this composition is close to the melting point of TiC (3073 ± 25 °C). During combustion of mixtures containing soot as a source of carbon, the products are ejected from the SHS wave [16]. In the present work, the stoichiometry of the mixture and the ball milling time were determined to enable combustion without any ejection effects. In the Ti+0.2C mixture, the SHS cannot be ignited, if the milling time is shorter than 2 min. However, the Ti+0.3C mixture milled for 1–10 min is combustible in both nitrogen and argon gases. The Ti+0.3C composition was selected for studying the ball milling effect on the phase composition of the SHS product.

**Figure 3:**
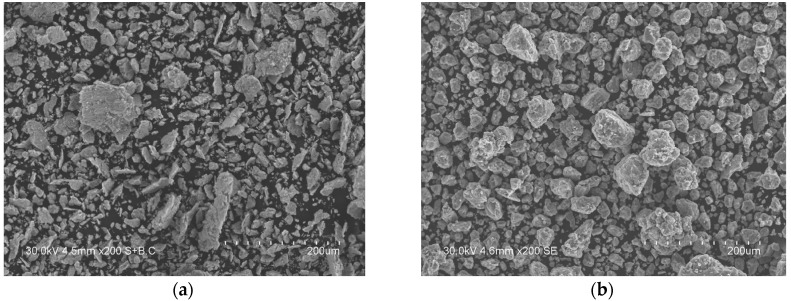
Morphology of mechanocomposite particles formed after 1 min (**a**), 10 min (**b**) of ball milling. The composition of the mixture is Ti+0.3C.

The morphological studies of the ball-milled powders showed that, at the early stages of milling, the size of titanium particles decreases, as they become agglomerated with the particles of carbon black. After 1 min of ball milling, mechanocomposites of platelet shape dominate in the mixture (Figure 3a). The morphology of the particles of the mixture milled for 10 min is shown in Figure 3b. It can be seen that the mechanocomposites have acquired the shape of equi-axed particles.

**Figure 4:**
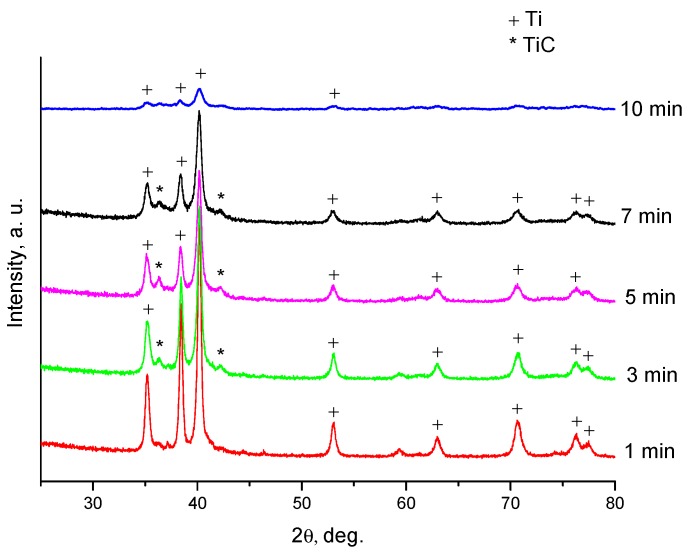
X-ray diffraction (XRD) patterns of the Ti+0.3C mixture ball-milled for 1, 3, 5, 7, and 10 min. The composition of the mixture is Ti+0.3C.

The XRD lines of titanium become broader as the time of ball milling increases (Figure 4). After 3 min of milling, titanium carbide starts forming, as indicated by the presence of reflections of this phase on the XRD patterns. The concentration of titanium carbide in the powder mixture increases with the milling time. The reaction is possible locally in the material volumes experiencing ball impacts during the treatment. The presence of the untransformed reactants in the ball-milled mixtures is confirmed by the mere fact that the mixtures are still combustible. Had a SHS reaction occurred during milling, it would have been impossible to ignite the ball-milled mixtures afterwards. 

**Figure 5:**
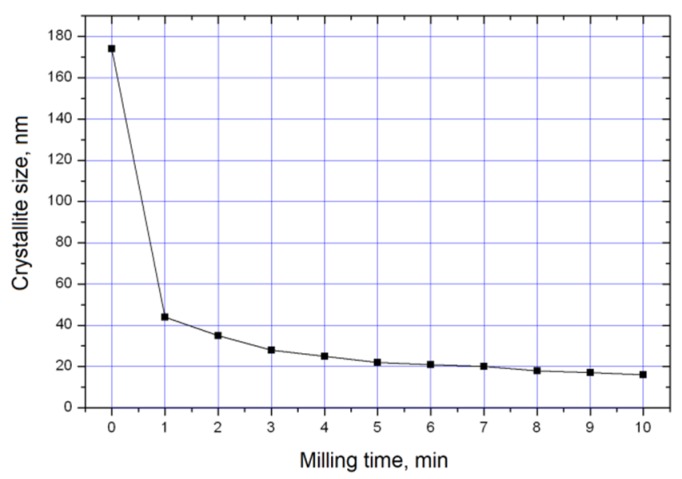
The dependence of the titanium crystallite size on the milling time of the Ti+0.3C powder mixture.

Figure 5 shows the dependence of the titanium crystallite size on the milling time of the Ti+0.3C mixture. After 1 min of milling, the crystallite size of titanium is greatly reduced as compared with the starting powder. Then, the size of crystallites decreases slowly with the milling time.

**Figure 6:**
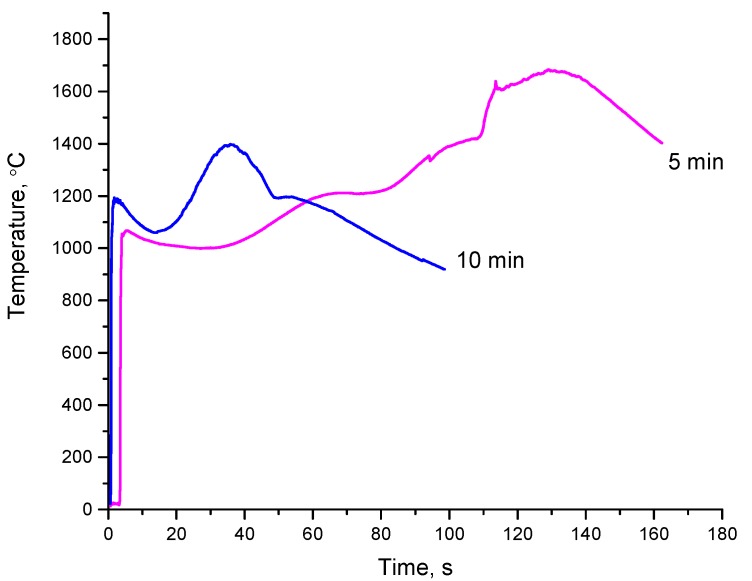
Thermograms of the SHS processes in the Ti+0.3C mixture ball-milled for 5 min and 10 min, combustion in nitrogen.

Figure 6 shows the thermograms of the combustion process of the Ti+0.3C mixture (ball-milled for 5 and 10 min) in nitrogen. It can be seen that the evolution of heat has a complex (multi-stage) character. As the first wave of SHS passes through the mixture milled for 5 min, the temperature increases up to 1070 ± 20 °C (the first stage of the synthesis). Then the temperature decreases down to ~1000 °C. After 30–35 s, the temperature increases slowly up to 1680 ± 15 °C (the second stage of the synthesis). At this moment, the sample shows a white glow. A similar multi-step combustion process was observed when granulated Ti+0.5C mixtures were subjected to combustion [21].

**Figure 7:**
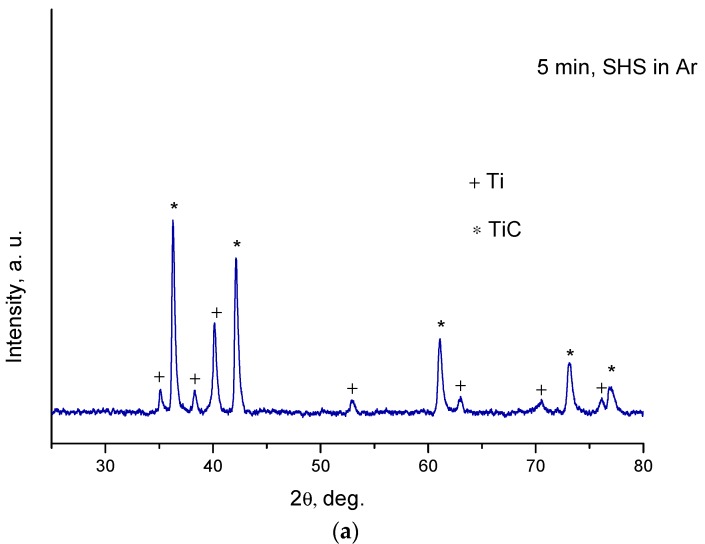

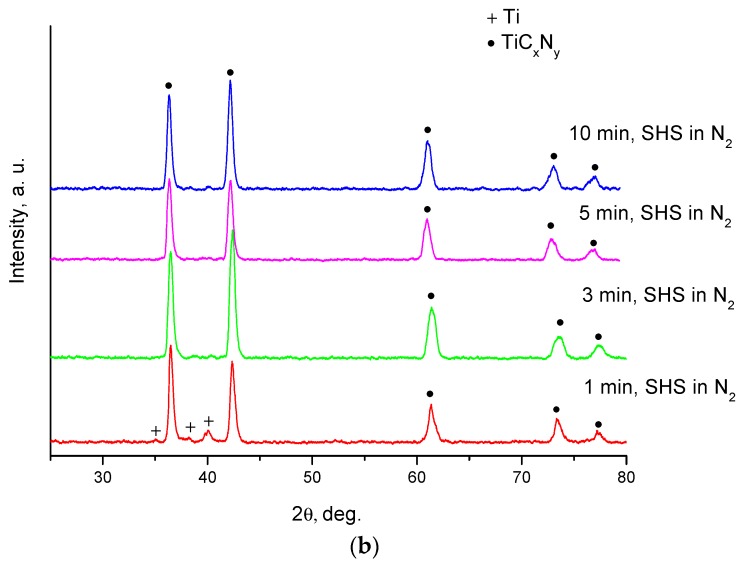
XRD patterns of the product of SHS in the Ti+0.3C mixture ball-milled for 5 min, combustion in argon (**a**), ball-milled for 1, 3, 5 and 10 min, combustion in nitrogen (**b**).

The products of combustion of the Ti+0.3C mixture in argon and nitrogen differ in their phase composition. The XRD phase analysis showed that the combustion product obtained in argon consisted of TiC and unreacted titanium (Figure 7a). The products obtained in nitrogen were single-phase when the reaction mixture was milled for a certain period of time, 5 min or longer (Figure 7b). Considering the phase composition of the SHS product, it can be assumed that an increase in the temperature of the sample at the second synthesis stage (Figure 6) is due to nitridation of the material after the passage of the first combustion wave. Consequently, combustion of the ball-milled Ti+0.3C mixtures in nitrogen is a multi-stage process. The first combustion wave passes quickly through the sample and is followed by slow nitridation of the material. The formation of titanium carbide is responsible for the first self-propagating reaction. 

The combustion rate of the Ti+0.3C mixtures changes with the milling time. As the milling time increases from 1 to 7 min, the combustion rate increases from 2.3 ± 0.2 to 11 ± 1 mm s^−1^. Combustion of powders ball-milled for 1–4 min is accompanied by the ejection of hot particles from the combustion wave. The SHS products obtained in nitrogen using mixtures milled for 1–4 min are composed of titanium carbonitride and metallic titanium. The intensities of the titanium lines on the XRD patterns of the combustion products obtained in nitrogen decrease as the milling time increases; the product obtained from a mixture ball-milled for 5 min is a single-phase carbonitride (Figure 7b). This sample shows no ejection of particles during the combustion. Samples milled for 1–7 min show similar thermograms. The duration of the nitridation stage decreases as the milling time of the mixtures increases; in the sample milled for 7 min, the nitridation occurs within 1 min. The mixture milled for 10 min demonstrates a combustion rate of 8 ± 1 mm s^−1^. The product of SHS conducted in this mixture is single-phase.

After 10 min of milling, the maximum combustion temperature decreases down to 1400 ± 20 °C. The duration of the nitridation stage decreases down to 30–40 s. The growth of the combustion rate with the milling time (in the 1–7 min range) can be, in part, due to a reduction of the crystallite size of titanium. As the milling time is increased from 7 min to 10 min, the crystallite size of titanium changes slightly (from 20 to 16 nm), while the concentration of titanium carbide in the ball-milled mixtures increases. The latter causes a reduction in the combustion rate of these mixtures.

**Figure 8:**
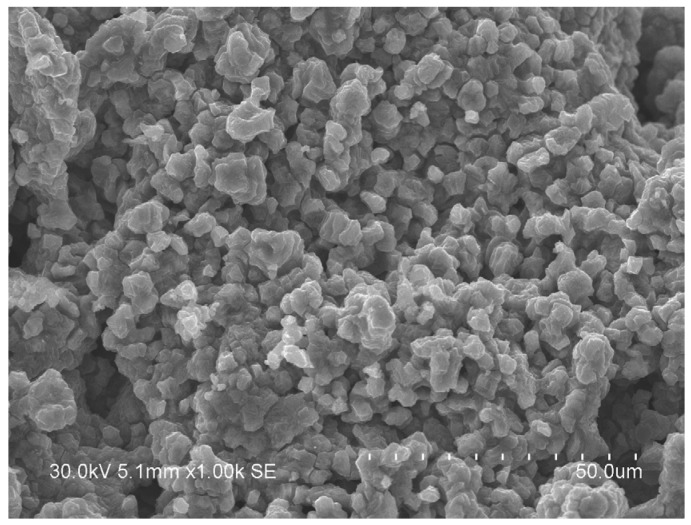
Micrograph of the product of SHS in the Ti+0.3C mixture ball-milled for 10 min. Combustion in nitrogen.

Figure 8 shows the morphology of the combustion products obtained in nitrogen from a mixture milled for 10 min. It can be seen that the product consists of particles 5–10 μm in size. These particles are composed of crystallites with a size of 60 ± 5 nm, as estimated from the XRD pattern of the product. As the maximum combustion temperature during SHS in this powder mixture is only 1400 ± 20 °C, solid-state combustion must have occurred (the temperatures of eutectics in the Ti-C and Ti-N_2_ systems are much higher). Our experiments show that the product of SHS is a single-phase carbonitride when the Ti+0.3C mixture is milled for durations equal to or longer than 5 min under the selected milling conditions.

In ref. [12], for the Ti+0.5C mixtures, a two-stage mechanism of the formation of titanium carbonitride by SHS in a nitrogen atmosphere is suggested. At the first stage, non-stoichiometric carbide TiC_0.5_ formed in the combustion wave. At the second stage, after the passage of the combustion wave, the carbide reacted with nitrogen to form TiC_0.5_N_0.5_. In the present work, in mixtures milled for 5 min or longer, after the first combustion wave, a TiC_x_-Ti composite forms that is capable of reacting with nitrogen to produce a single-phase carbonitride as a result of SHS.

We have found that there exist possibilities to change the composition of the carbonitride phases synthesized by SHS in terms of carbon and nitrogen content. One of these possibilities is to increase the concentration of carbon in the starting mixtures. The second way to change the composition of the carbonitride is to add previously synthesized TiC to the reaction mixture. Further research should be aimed to elucidate the detailed mechanism of combustion of ball-milled mixtures and the influence of the fine structure of the mixture on the processes of gas/solid interaction.

## 4. Conclusions

In this work, the influence of ball milling of the phase composition of the products of SHS conducted in Ti+0.3C powder mixtures in a nitrogen atmosphere was studied. The key result of this work is the formation of titanium carbonitride at a pressure of nitrogen equal to atmospheric pressure, which became possible when high-energy ball-milled reaction mixtures were used. Titanium–carbon black mixtures must be milled for a certain period of time for the combustion synthesis to give a single-phase titanium carbonitride product.
